# *QuickStats:* Percentage* of Adults Who Used the Internet in the Past 12 Months to Communicate with a Doctor or Doctor's Office,^†^ by Urbanization Level^§^ — National Health Interview Survey, United States, July–December 2022^¶^

**DOI:** 10.15585/mmwr.mm7244a5

**Published:** 2023-11-03

**Figure:**
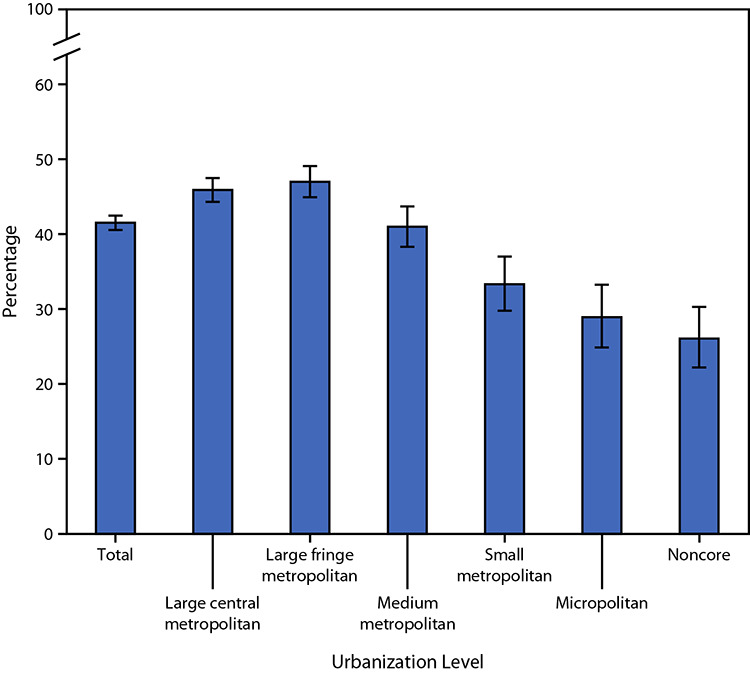
During July–December 2022, 41.5% of U.S. adults used the Internet in the past 12 months to communicate with a doctor or doctor’s office. The percentage of adults who used the Internet to communicate with a doctor or doctor's office was highest among adults living in large central metropolitan (45.9%) and large fringe metropolitan (47.0%) counties, then decreased with decreasing level of urbanization to 26.1% for those living in noncore counties.

**Source:** National Center for Health Statistics, National Health Interview Survey, 2022. https://www.cdc.gov/nchs/nhis.htm

